# Multidisciplinary Management of Neuroendocrine Neoplasia: A Real-World Experience from a Referral Center

**DOI:** 10.3390/jcm8060910

**Published:** 2019-06-25

**Authors:** Ludovica Magi, Federica Mazzuca, Maria Rinzivillo, Giulia Arrivi, Emanuela Pilozzi, Daniela Prosperi, Elsa Iannicelli, Paolo Mercantini, Michele Rossi, Patrizia Pizzichini, Andrea Laghi, Alberto Signore, Paolo Marchetti, Bruno Annibale, Francesco Panzuto

**Affiliations:** 1 Digestive Disease Unit, ENETS Center of Excellence, Sant’Andrea University Hospital, 00189 Rome, Italy; ludovicamagi@hotmail.it (L.M.); mariarinzivillo@gmail.com (M.R.); Bruno.annibale@uniroma1.it (B.A.); 2 Medical Oncology Unit, ENETS Center of Excellence, Sant’Andrea University Hospital, 00189 Rome, Italy; federica.mazzuca@uniroma1.it (F.M.); giulia.arrivi@uniroma1.it (G.A.); Paolo.marchetti@uniroma1.it (P.M.); 3 Department of Clinical and Molecular Medicine, “Sapienza” University of Rome, 00189 Rome, Italy; Emanuela.pilozzi@uniroma1.it; 4 Pathologic Anatomy and Molecular Morphology Unit, ENETS Center of Excellence, Sant’Andrea University Hospital, 00189 Rome, Italy; 5 Nuclear Medicine Unit, ENETS Center of Excellence, Sant’Andrea University Hospital, 00189 Rome, Italy; Dprosperi@ospedalesantandrea.it (D.P.); ppizzichini@ospedalesantandrea.it (P.P.); alberto.signore@uniroma1.it (A.S.); 6 Radiology Unit, ENETS Center of Excellence, Sant’Andrea University Hospital, 00189 Rome, Italy; Elsa.iannicelli@uniroma1.it (E.I.); Michele.rossi@uniroma1.it (M.R.); Andrea.laghi@uniroma1.it (A.L.); 7 Department of Medical-Surgical Sciences and Translational Medicine, “Sapienza” University of Rome, 00189 Rome, Italy; Paolo.mercantini@uniroma1.it; 8 Surgery Unit, ENETS Center of Excellence, Sant’Andrea University Hospital, 00189 Rome, Italy

**Keywords:** neuroendocrine tumors, multidisciplinary, management, outcome, grading, staging

## Abstract

Purpose: Multidisciplinary approach is widely advised for an effective care of patients with neuroendocrine neoplasia (NEN). Since data on efficacy of multidisciplinary management of NENs patients in referral centers are scanty, this study aimed at analyzing the modality of presentation and clinical outcome of patients with NENs managed by a dedicated multidisciplinary team. Methods. In this prospective observational study, we included all consecutive new patients visiting the Sant’Andrea Hospital in Rome (ENETS—Center of Excellence) between January 2014 and June 2018. Results. A total of 195 patients were evaluated. The most frequent sites were pancreas (38.5%), small bowel (22%), and lung (9.7%). Median Ki67 was 3%. After the first visit at the center, additional radiological and/or nuclear medicine procedures were requested in 163 patients (83.6%), whereas histological data revision was advised in 84 patients (43.1%) (revision of histological slides: 27.7%, new bioptic sampling: 15.4%). After that, disease imaging staging and grading was modified in 30.7% and 17.9% of patients, respectively. Overall, a change in therapeutic management was proposed in 98 patients (50.3%). Conclusions. Multidisciplinary approach in a dedicated team may lead to change of disease imaging staging and grading in a significant proportion of patients. Enhancing referral routes to dedicated-NEN center should be promoted, since it may improve patients’ clinical outcome.

## 1. Introduction

Neuroendocrine neoplasia (NEN) is a group of rare and heterogenous diseases, in terms of both pathological and clinical features. Their prognosis is affected by several factors, including primary tumor site, staging, and grading [1,2,3]. They promise a clinical challenge for physicians, because they may have various growth patterns ranging from very slowly progressive to rapidly aggressive tumors. An effective diagnosis of NEN is based on clinical presentation, pathology, cross-sectional imaging (computed tomography (CT) or magnetic resonance imaging (MRI)), and functional nuclear medicine procedures, including 68-Gallium PET and 18FDG PET [4,5,6]. Recently, the involvement of the immune system and the role of tumor micro-environment has also been suggested as important in tumor evolution [7]. Surgery is widely considered the sole chance to cure patients; however, it is often not feasible due to advanced metastatic disease at time of diagnosis. In these patients, for whom medical treatment is required, several therapeutic options are available, including somatostatin analogs (octreotide and lanreotide), peptide receptor radionuclide therapy (PRRT), targeted therapies (everolimus and sunitinib), and systemic chemotherapy [4].

Due to the complexity of NEN management, a multidisciplinary approach is widely advised for an effective care of patients with this uncommon kind of cancer. Multidisciplinary care is strongly encouraged by both the European and North American Neuroendocrine Tumor Society [8,9]. There has been some evidence of better survival in patients managed in centers with dedicated multidisciplinary team (MDT) compared to those treated with standard care in different kinds of cancers [10,11]. However, the real impact of MDT on patients’ survival may vary depending on structural and functional components and the expertise of the participants [12]. Since data on the efficacy of multidisciplinary management of patients with NENs in specialized centers with dedicated MDT are scanty, this study aimed at analyzing the modalities of presentation and clinical outcome of patients with NENs managed in a center of excellence with a dedicated MDT.

## 2. Patients and Methods

This is a prospective observational study including all consecutive new patients visiting the Sant’Andrea Hospital site of Rome (part of the Rome ENETS Center of Excellence) between January 2014 and June 2018. In accordance with the center standard of procedures, all major clinical and pathological data were collected in a computer anonymized database. All patients were discussed in an NEN multidisciplinary team that included several clinicians involved in patients’ management: oncologist, gastroenterologist, surgeon, nuclear medicine physician, radiologist, and pathologist.

Based on data retrieved from available charts, gastrointestinal and pancreatic NENs were retrospectively classified according to WHO 2010 [13] and WHO 2017 [14] classifications, whereas the WHO 2014 [15] classification was used for lung NENs. Tumor grading was assessed according to the ENETS grading system in gastro-entero-pancreatic (GEP) NENs, as well as in lung NENs [1,16,17]. Pathological revision was performed in those patients for whom available histological information was not accurate enough to obtain an NEN diagnosis in accordance with ENETS standards of care [18]. When required, repeating bioptic sampling was proposed to the patients after MDT discussion. Patients’ follow-up was performed in accordance with ENETS recommendations [19].

The distribution of continuous variables was reported as the median and interquartile range (IQR; 25th–75th percentiles) or range, as appropriate. A comparison between the subgroups was carried out using Fisher’s exact test or the chi-square test for noncontinuous variables, whereas the Mann–Whitney U test was used to compare the non-normally distributed continuous independent variables, as appropriate. Overall survival analysis was performed using the Kaplan–Meier method. This work was carried out in accordance with the Declaration of Helsinki. Full informed consent for data collection was obtained from all patients.

## 3. Results

**Figure 1:**
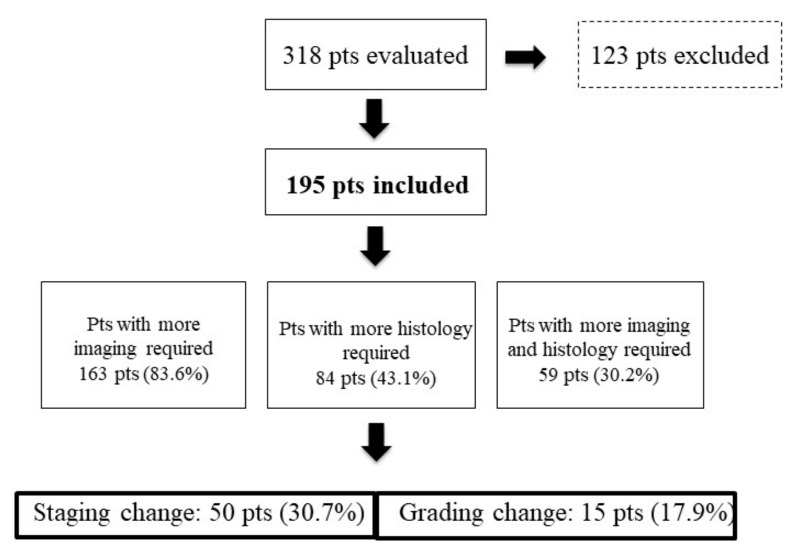
Staging and grading modification after visit at the Center. pts, patients.

A total of 318 patients were evaluated. Of these, 123 patients (38.7%) were excluded because they had been referred to the center with the intention of obtaining a second opinion (Figure 1); since these patients were not taken in care by the center, no data on their follow-up were available. Thus, final analysis was performed on 195 patients, including 94 males (48.2%), with a median age of 59 years (IQR 51–70.5 years). Of these, 163 patients had GEP NENs (83.6%) and 19 patients had lung primary NEN (9.7%). In the remaining 13 patients (6.7%), the primary tumor site was unknown (Table 1).

At time of initial visit at the center, the Ki67 value was available in 177 patients (90.8%), the median value being 3% (IQR 2–9). All but 7 patients (96.4%) had tumors with well differentiated morphology.

Overall, 147 patients (75.4%) already had NEN diagnosis at time of referral; in these patients, the median interval between initial NEN diagnosis and time of referral to the center was 4 months (IQR 2–13.5 months). The remaining 48 patients (24.6%) were newly diagnosed at the center. Patients’ general features are summarized in Table 1. Overall, 68 patients (34.8%) were discussed multiple times by MDT after initial evaluation.

A total of 63 patients (32.3%) got in touch with the center using the center’s website form, whereas 132 patients (67.7%) booked the first visit through public health regional system tools (dedicated phone number, direct hospital access). Seventy-four patients (37.9%) were referred to the center by other hospitals. The median waiting time to obtain the first visit in the NEN-dedicated ambulatory was 7 days (IQR 7–10 days).

### Patients’ Management

After first visit, additional cross-sectional radiological examinations and/or nuclear medicine diagnostic procedures were requested in 163 patients (83.6%) (Figure 1). In particular, CT or MRI was prescribed in 123 patients (63.1%) (additional CT or MR was considered to be necessary, because either a new updated staging was necessary or CT/MR had not been previously performed or they were of insufficient image quality or incomplete according to the imaging standard of our center), ^68^Ga-DOTA-NOC Positron Emission Tomography (PET)/CT in 107 patients (54.9%) (resulting positive in 83 of them, 77.7%), and (^18^F)FDG PET/CT in 42 patients (21.5%) (resulting positive in 15 of them, 35.7%). Dual PET/CT,with ^68^Ga-DOTA-NOC and (^18^F) fluorodeoxyglucose (FDG), were performed in 21 patients (10.8%). Overall, a positive finding was observed in 65% of patients for whom an additional functional imaging procedure (^68^Ga-DOTA-NOC or (^18^F)FDG-PET) was requested.

After evaluating the requested radiological/nuclear medicine procedures, a change in disease staging was performed in 50/163 patients (30.7%).

Integration of available pathological data was advised in 84 patients (43.1%) (Figure 1). Specifically, revision of available histological slides was required in 54 patients (27.7%), whereas new bioptic sampling was performed in 30 patients (15.4%). Pathological revision consisted of histology in all but 2 patients, in whom cytology was performed. After histological data integration, pathological change in terms of grading modification was observed in 15 patients (17.9%). Specifically, a grading increase was observed in 10 patients (5 patients moved from G1 to G2, 5 patients from G2 to G3), whereas a grading decrease was observed in the remaining 5 patients (from G2 to G1).

A total of 174 patients (89.2%) received a decision concerning subsequent follow-up within 1 month after their initial visit at the center. All suggestions proposed by the MDTs were executed. Overall, a change in clinical management was proposed in 98 patients (50.3%). Of these, 67 patients (68.4%) received medical treatment (changes in medical treatments after first MDT discussion are detailed in Table 2) (most frequently somatostatin analogs (37 patients, 37.8%); followed by everolimus (15 patients, 15.3%), systemic chemotherapy (6 patients, 6.1%), sunitinib (5 patients, 2.7%), and peptide receptor radionuclide therapy (4 patients 4%). Nine patients (9.2%) underwent surgery, and 19 patients (19.4%) were followed up without medical or surgical intervention.

A total of 28 patients (14.4%) died of disease during a median follow-up period of 17 months (IQR 7.2–33 months) after initial diagnosis at the center. Median survival after initial diagnosis at the center was not reached, whereas 5-y survival rate was 62.6%. Median survival in stage IV patients was 59 months.

## 4. Discussion

Although several studies have demonstrated a potential positive impact on patients’ clinical care in different kinds of cancers, it has been recently suggested that tumor boards are only as good as their structural and functional components and the expertise of the participants [12]. As far as NENs are concerned, knowledge of MDT impact on patients’ care is even scantier [20,21,22]. International guidelines for NENs emphasize collaboration among diverse medical disciplines to improve patients’ care and standardize diagnostic and therapeutic approaches. However, despite the widespread use of multidisciplinary teams for the management of NEN patients in the clinical practice, few data on their effect on care exist.

The present study reports the real-world experience of a referral center in which, according with the ENETS standard of procedures, newly patients are routinely discussed in a multidisciplinary setting. Interestingly, almost 2/3 of patients included presented with advanced disease at time of initial referral, stage 3 and 4 being observed in 19.5% and 45.1%, respectively, or with tumor with moderate-high proliferative activity, with the G2 and G3 group representing 41% and 13.9%, respectively. In accordance with other series [23,24], this figure confirms that NEN patients presenting to a referral center often have advanced, progressive disease requiring specific diagnostic investigations and tailored therapeutic approaches that need to be shared in a multidisciplinary discussion.

In the present study, most patients (75.4%) referred to the center with NEN had already been diagnosed at the time of center referral, with the median interval between initial diagnosis and center referral being 4 months (IQR 2–13.5). Almost all patients (89.2%) received decision concerning subsequent follow-up within 1 month after their initial visit. Prompt multidisciplinary evaluation helps to expedite the beginning of optimal therapeutic strategy in NEN patients, which may result in a more favorable clinical outcome. Recent studies reported a long interval varying from 24 to 53.8 months from onset of symptoms and definitive diagnosis in NEN patients, leading to a delayed diagnosis and a plausible worse overall prognosis [25]. Early referral to an NEN-dedicated center may give patients a higher probability to receive prompt accurate disease staging, a tailored therapeutic approach, and may result in a better chance to participate in clinical trials, an option which is considered the best management opportunity to be especially encouraged (NCCN Guidelines, www.nccn.org).

After referral to the center, integration or revision of pathological data was advised in a significant proportion of patients (43.1%), because available data were considered not accurate enough in accordance with the ENETS standards of care [18], with new bioptic sampling being advised in 15.4% of patients. In accordance with data obtained by pathological data integration/revision, a grading change occurred in 17.9% of patients. It is well known that grading is the most powerful prognostic factor in NENs and may be considered a decision-driving marker when planning treatment [2,3,26,27,28]. Tumor grading needs to be assessed by evaluating the Ki67 proliferation index, and the number of counted cells (recommended 500 to 2000) has to be mentioned [29]. Clinicians dealing with NEN patients should always check whether the pathology report includes an accurate grading assessment, otherwise pathological data revision or integration by repeating tumor biopsy is advocated. Since there is the possibility of Ki67 changes throughout the disease course [30], repeating biopsy has also been proposed in those patients presenting with progressive disease, since it might help with planning an appropriate clinical management and therapeutic approach [31].

Additional imaging procedures were advised in the majority of patients (83.6%) after referral to the center. Interestingly, somatostatin receptor imaging (SRI) with ^68^Ga-peptides was required in more than half of the patients (54.9%), a figure that highlights the role of this technique in the management of NEN patients. To date, SRI is considered the most effective diagnostic tool in NENs. Performing SRI may result in a change in clinical management in up to 45% of NEN patients [32], particularly due to the high ability of this technique to detect distant extra-hepatic metastases [33], whose presence is known to be a strong negative prognostic factor affecting patients’ clinical outcome [34]. Changes in clinical management after multidisciplinary discussion consisted of surgical treatment in a relatively low proportion of patients (9%), although 35.4% of include patients have limited disease (Stage I–II). This figure may be due to different reasons, including the presence of patients who had been already operated on before being referred to the center, and the inclusion of patients who rarely require surgical treatment (i.e., type I gastric NENs and small rectal NENs). The present study shows that in a real-world setting of a NEN referral center, investigating tumor somatostatin receptor expression by additional SRI and advising additional histological data through the revision of available pathological data or repeating tumor biopsy are considered mandatory steps before planning patients’ management. However, this study has some limitations: i. a significant proportion of patients (38.7%) were excluded from the final analysis due to the lack of relevant data, since these patients were referred to the center with the intention of obtaining a second opinion and were not followed-up; ii. the population enrolled was relatively heterogeneous, including primary tumors raising from different sites (i.e., GEP and lung); iii. The decision to request additional imaging procedures or histological evaluation was made on a case by case basis by the MDT without a specific decision-making predefined protocol; iv. data on tumor markers (i.e., Chromogranin A) were available in a minority of patients and were thus not reported in the final analysis.

## 5. Conclusions

A multidisciplinary approach offers the best prospect for planning optimal management and improving clinical outcomes in patients with NENs. Early referral to NEN-dedicated centers may shorten delay in diagnosis and increase the opportunity for patients to receive the best care in terms of follow-up and therapeutic approach. Enhancing referral routes to NEN-dedicated centers with experienced MDTs should be promoted, since it may improve patients’ clinical outcome.

## Figures and Tables

**Table 1:** Patients’ general features.

	Overall *n* = 195	Newly Diagnosed *n* = 48	Referred *n* = 147	*p*-Value
Primary site				
Pancreas	75 (38.5%)	22 (45.8%)	53 (36%)	0.642
Small bowel	43 (22.1%)	10 (20.8%)	33 (22.4%)	
Rectum	10 (5.1%)	2 (4.2%)	8 (5.4%)	
Appendix	9 (4.6%)	2 (4.2%)	7 (4.7%)	
Lung	19 (9.7%)	6 (12.5%)	13 (8.8%)	
Other	39 (20%)	6 (12.5%)	33 (22.4%)	
Grading				
G1	88 (45.1%)	22 (45.8%)	66 (44.9%)	0.053
G2	80 (41%)	18 (37.5%)	62 (42.2%)	
G3	27 (13.9%)	8 (16.7%)	19 (12.9%)	
Median Ki67 (IQR, range)	3% (2–9, 1–90)	2% (2–5, 1–40)	3% (2–10, 1–90)	0.212
Staging				
Stage 1	42 (21.5%)	13 (27.1%)	29 (19.7%)	0.080
Stage 2	27 (13.9%)	6 (12.5%)	21 (14.3%)	
Stage 3	38 (19.5%)	8 (16.7%	30 (20.4%)	
Stage 4	88 (45.1%)	21 (43.7%)	67 (45.6%)	

**Table 2:** Changes in medical treatments after first multidisciplinary discussion.

	Before MDT *	After MDT
Somatostatin analogs	23 (11.8%)	37 (19%)
Targeted therapies	3 (1.5%)	20 (10.3%)
Peptide receptor radionuclide therapy	1 (0.5%)	4 (2%)
Systemic chemotherapy	26 (13.3%)	6 (3%)

* 14/67 patients were not receiving medical treatment before discussion.
